# Supplementary material for: A Molecular Signature Determines the Prognostic and Therapeutic Subtype of Non-Muscle-Invasive Bladder Cancer Responsive to Intravesical Bacillus Calmette-Guérin Therapy
Source: Int J Mol Sci. 2021 Feb 1;22(3):1450. doi: 10.3390/ijms22031450 (PMC7867154; doi:10.3390/ijms22031450)
Supplement: Supplementary file 1 [file ijms-22-01450-s001.zip › Figure_S1.pptx]

## Slide 1
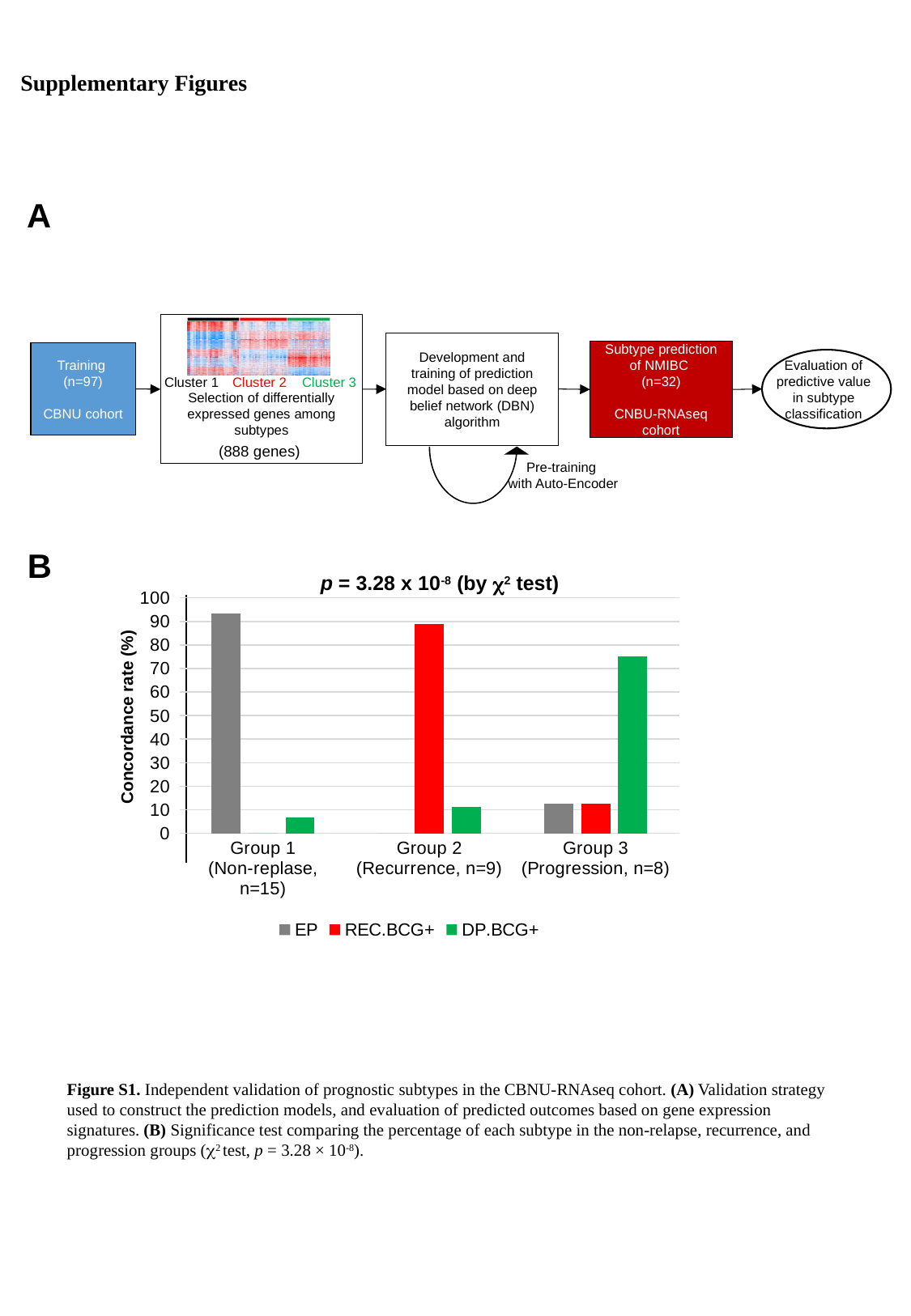

Supplementary Figures
A
Selection of differentially expressed genes among subtypes
Development and training of prediction model based on deep belief network (DBN) algorithm
Subtype prediction of NMIBC
(n=32)
CNBU-RNAseq cohort
Training
(n=97)
CBNU cohort
Evaluation of predictive value in subtype classification
Cluster 1
Cluster 2
Cluster 3
(888 genes)
Pre-training
with Auto-Encoder
B
p = 3.28 x 10^-8^ (by χ2 test)
### Chart
| Category | EP | REC.BCG+ | DP.BCG+ |
|---|---|---|---|
| Group 1
(Non-replase, n=15) | 93.33333333333334 | 0.0 | 6.666666666666667 |
| Group 2
(Recurrence, n=9) | 0.0 | 88.88888888888889 | 11.11111111111111 |
| Group 3
(Progression, n=8) | 12.5 | 12.5 | 75.0 |Concordance rate (%)
Figure S1. Independent validation of prognostic subtypes in the CBNU-RNAseq cohort. (A) Validation strategy used to construct the prediction models, and evaluation of predicted outcomes based on gene expression signatures. (B) Significance test comparing the percentage of each subtype in the non-relapse, recurrence, and progression groups (χ2 test, p = 3.28 × 10^-8^).
