# Supplementary material for: A Molecular Signature Determines the Prognostic and Therapeutic Subtype of Non-Muscle-Invasive Bladder Cancer Responsive to Intravesical Bacillus Calmette-Guérin Therapy
Source: Int J Mol Sci. 2021 Feb 1;22(3):1450. doi: 10.3390/ijms22031450 (PMC7867154; doi:10.3390/ijms22031450)
Supplement: Supplementary file 1 [file ijms-22-01450-s001.zip › Figure_S2.pptx]

## Slide 1
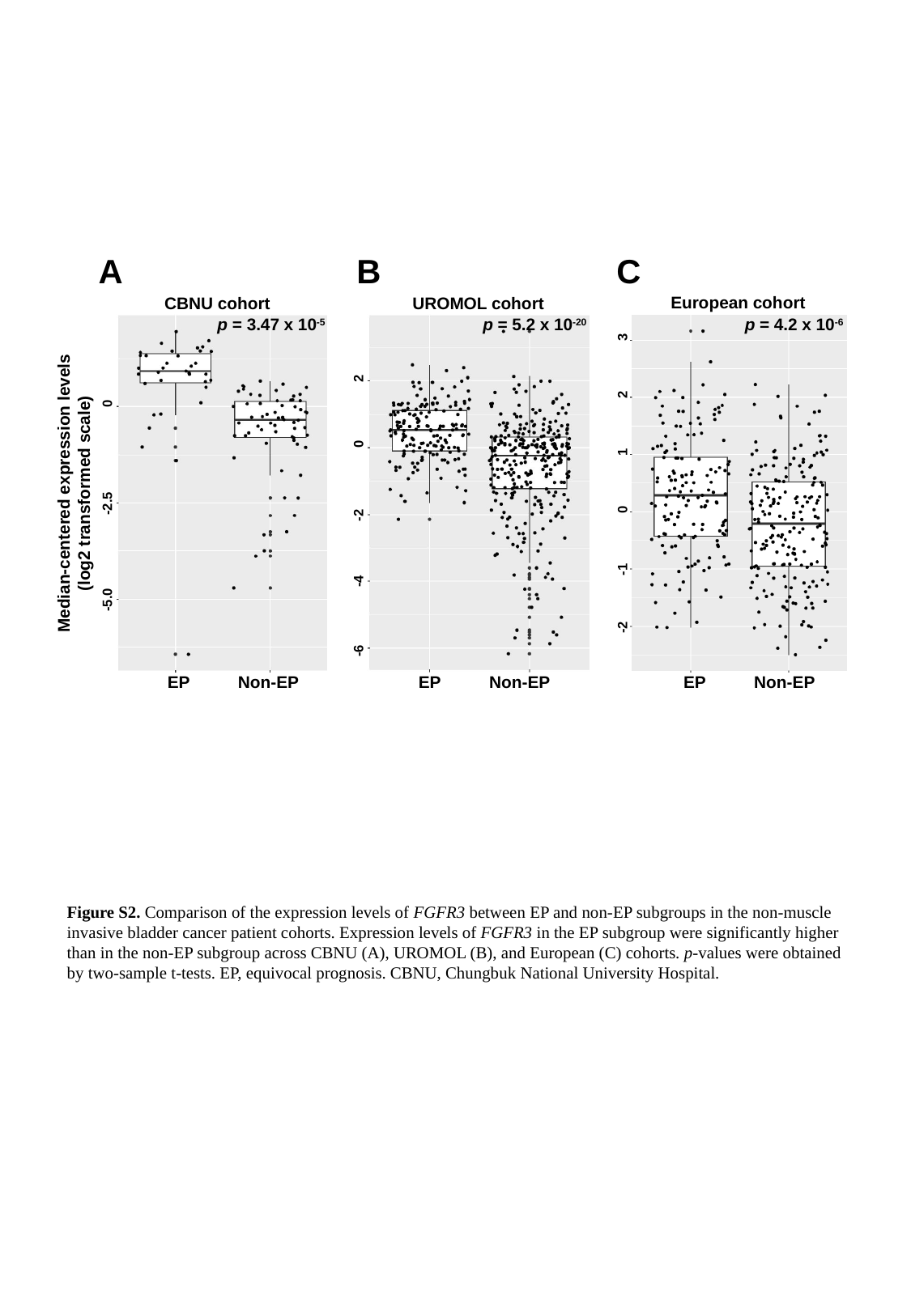

A
B
C
European cohort
UROMOL cohort
CBNU cohort
p = 3.47 x 10^-5^
p = 5.2 x 10^-20^
p = 4.2 x 10^-6^
-5.0 -2.5 0
Median-centered expression levels
(log2 transformed scale)
 -2 -1 0 1 2 3
 -6 -4 -2 0 2
EP
Non-EP
EP
Non-EP
EP
Non-EP
Figure S2. Comparison of the expression levels of FGFR3 between EP and non-EP subgroups in the non-muscle invasive bladder cancer patient cohorts. Expression levels of FGFR3 in the EP subgroup were significantly higher than in the non-EP subgroup across CBNU (A), UROMOL (B), and European (C) cohorts. p-values were obtained by two-sample t-tests. EP, equivocal prognosis. CBNU, Chungbuk National University Hospital.
