# Supplementary material for: A Molecular Signature Determines the Prognostic and Therapeutic Subtype of Non-Muscle-Invasive Bladder Cancer Responsive to Intravesical Bacillus Calmette-Guérin Therapy
Source: Int J Mol Sci. 2021 Feb 1;22(3):1450. doi: 10.3390/ijms22031450 (PMC7867154; doi:10.3390/ijms22031450)
Supplement: Supplementary file 1 [file ijms-22-01450-s001.zip › Figure_S3.pptx]

## Slide 1
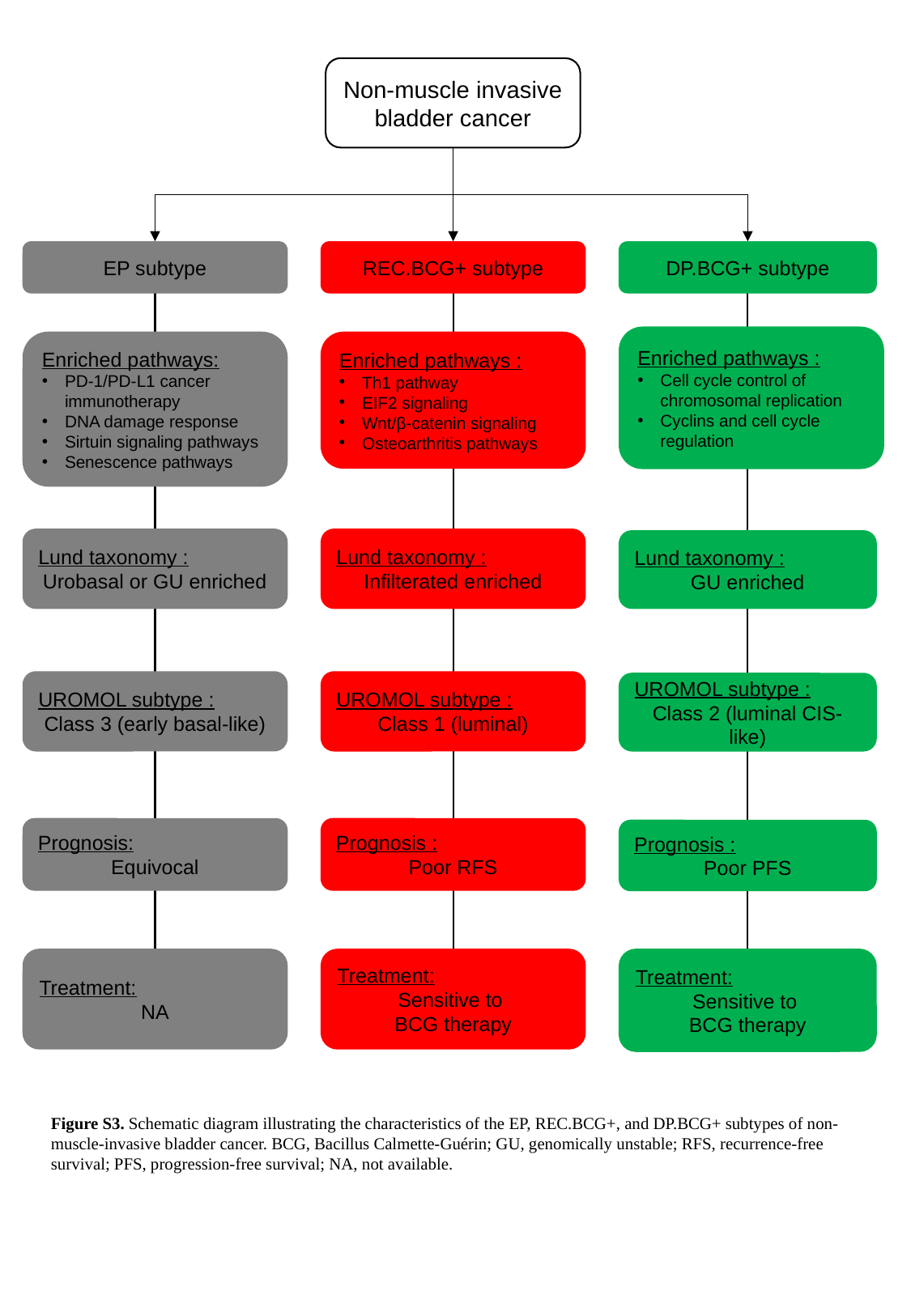

Non-muscle invasive bladder cancer
EP subtype
REC.BCG+ subtype
DP.BCG+ subtype
Enriched pathways :
Cell cycle control of chromosomal replication
Cyclins and cell cycle regulation
Enriched pathways :
Th1 pathway
EIF2 signaling
Wnt/β-catenin signaling
Osteoarthritis pathways
Enriched pathways:
PD-1/PD-L1 cancer immunotherapy
DNA damage response
Sirtuin signaling pathways
Senescence pathways
Lund taxonomy :
Urobasal or GU enriched
Lund taxonomy :
Infilterated enriched
Lund taxonomy :
GU enriched
UROMOL subtype :
Class 3 (early basal-like)
UROMOL subtype :
Class 1 (luminal)
UROMOL subtype :
Class 2 (luminal CIS-like)
Prognosis:
Equivocal
Prognosis :
Poor RFS
Prognosis :
Poor PFS
Treatment:
NA
Treatment:
Sensitive to
BCG therapy
Treatment:
Sensitive to
BCG therapy
Figure S3. Schematic diagram illustrating the characteristics of the EP, REC.BCG+, and DP.BCG+ subtypes of non-muscle-invasive bladder cancer. BCG, Bacillus Calmette-Guérin; GU, genomically unstable; RFS, recurrence-free survival; PFS, progression-free survival; NA, not available.
