# Supplementary material for: A Molecular Signature Determines the Prognostic and Therapeutic Subtype of Non-Muscle-Invasive Bladder Cancer Responsive to Intravesical Bacillus Calmette-Guérin Therapy
Source: Int J Mol Sci. 2021 Feb 1;22(3):1450. doi: 10.3390/ijms22031450 (PMC7867154; doi:10.3390/ijms22031450)
Supplement: Supplementary file 1 [file ijms-22-01450-s001.zip › Figure_S4.pptx]

## Slide 1
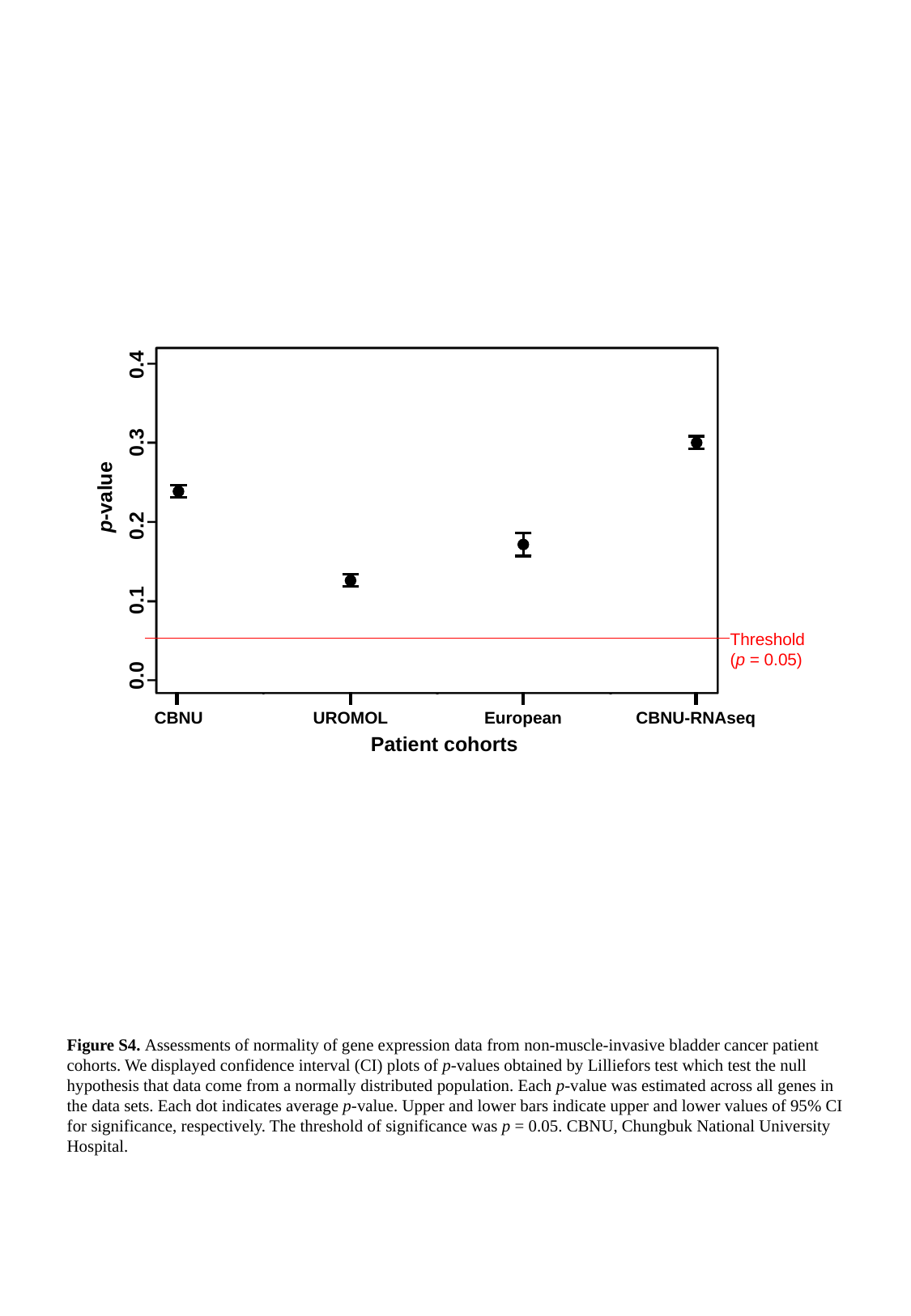

0.4
0.3
p-value
0.2
0.1
Threshold
(p = 0.05)
0.0
CBNU
UROMOL
European
CBNU-RNAseq
Patient cohorts
Figure S4. Assessments of normality of gene expression data from non-muscle-invasive bladder cancer patient cohorts. We displayed confidence interval (CI) plots of p-values obtained by Lilliefors test which test the null hypothesis that data come from a normally distributed population. Each p-value was estimated across all genes in the data sets. Each dot indicates average p-value. Upper and lower bars indicate upper and lower values of 95% CI for significance, respectively. The threshold of significance was p = 0.05. CBNU, Chungbuk National University Hospital.
