# Supplementary material for: A Molecular Signature Determines the Prognostic and Therapeutic Subtype of Non-Muscle-Invasive Bladder Cancer Responsive to Intravesical Bacillus Calmette-Guérin Therapy
Source: Int J Mol Sci. 2021 Feb 1;22(3):1450. doi: 10.3390/ijms22031450 (PMC7867154; doi:10.3390/ijms22031450)
Supplement: Supplementary file 1 [file ijms-22-01450-s001.zip › Table_S1.docx]

**Table S1.** Baseline characteristics of NMIBC patient cohorts.

| **Variables** | **CBNU**  **cohort** | **UROMOL cohort** | **European**  **cohort** | **CBNU-RNAseq cohort** |
| --- | --- | --- | --- | --- |
| Patients (n) | 103 | 460 | 353 | 32 |
| Gender, n (%) |  |  |  |  |
| Male | 87 (84.5) | 357 (77.6) | 282 (79.9) | 26 (81.3) |
| Female | 16 (15.5) | 103 (22.4) | 68 (19.3) | 6 (18.8) |
| NA |  |  | 3 (0.8) |  |
| Age (years) |  |  |  |  |
| Median | 66 | 69 | 69 | 72 |
| Range | 24-88 | 23-96 | 27-95 | 24–81 |
| Stage, n (%) |  |  |  |  |
| Ta | 23 (22.3) | 345 (75) | 188 (53.3) | 10 (31.3) |
| T1 | 80 (77.7) | 112 (24.3) | 162 (45.9) | 22 (68.8) |
| Other |  | 3 (0.7) | 3 (0.8) |  |
| Grade (1973 system), n (%) |  |  |  |  |
| Grade 1 | 29 (28.2) |  |  |  |
| Grade 2 | 57 (55.3) |  |  |  |
| Grade 3 | 17 (16.5) |  |  |  |
| Grade (2004 system), n (%) |  |  |  |  |
| PUNLMP |  | 7 (1.5) | 33 (9.3) |  |
| Low |  | 277 (60.2) | 97 (27.5) | 21 (65.6) |
| High |  | 176 (38.3) | 223 (63.2) | 11 (34.4) |
| CIS |  |  |  |  |
| No | 103 (100) | 389 (84.6) | 302 (85.6) | 32 (100) |
| Yes |  | 71 (15.4) | 51 (14.4) |  |
| Intravesical Therapy, n (%) |  |  |  |  |
| No | 47 (45.6) | 372 (80.9) | 263 (74.5) | 2 (6.3) |
| Yes | 56 (54.4) | 88 (19.1) | 90 (25.5) | 30 (93.8) |
| Median follow-up (months) | 71.3 | 33 | 52 | 75.4 |
| Abbreviations: NMIBC, non-muscle invasive bladder cancer; CBNU, Chungbuk National University Hospital; NA, not available | | | | |
