# Supplementary material for: A Molecular Signature Determines the Prognostic and Therapeutic Subtype of Non-Muscle-Invasive Bladder Cancer Responsive to Intravesical Bacillus Calmette-Guérin Therapy
Source: Int J Mol Sci. 2021 Feb 1;22(3):1450. doi: 10.3390/ijms22031450 (PMC7867154; doi:10.3390/ijms22031450)
Supplement: Supplementary file 1 [file ijms-22-01450-s001.zip › Table_S2.docx]

**Table S2.** Comparisons of EORTC risk scores among prognostic subgroups in the CBNU-RNAseq cohort.

|  | **Group 1 (n=15)**  **(no recurrence or progression)** | **Group 2 (n=9)**  **(Recurrence)** | **Group 3 (n=8)**  **(Progression)** | ***p*-value*** |
| --- | --- | --- | --- | --- |
| **EORTC 1 year Recurrence Prediction score** | | | | 0.413 |
| **15% risk** | 0 | 0 | 0 |  |
| **24% risk** | 7 (46.7) | 4 (44.4) | 2 (40.6) |  |
| **38% risk** | 8 (53.3) | 5 (55.6) | 5 (56.3) |  |
| **61% risk** | 0 | 0 | 1 (3.1) |  |
| **EORTC 5 year Recurrence Prediction score** | | | | 0.413 |
| **31% risk** | 0 | 0 | 0 |  |
| **46% risk** | 7 (46.7) | 4 (44.4) | 2 (40.6) |  |
| **62% risk** | 8 (53.3) | 5 (55.6) | 5 (56.3) |  |
| **78% risk** | 0 | 0 | 1 (3.1) |  |
| **EORTC 1 year Progression Prediction score** | | | | 0.716 |
| **0.2% risk** | 1 (6.7) | 2 (22.2) | 0 |  |
| **1% risk** | 4 (26.7) | 2 (22.2) | 4 (50.0) |  |
| **5% risk** | 10 (66.7) | 5 (55.6) | 4 (50.0) |  |
| **17% risk** | 0 | 0 | 0 |  |
| **EORTC 5 year Progression Prediction score** | | | | 0.716 |
| **0.8% risk** | 1 (6.7) | 2 (22.2) | 0 |  |
| **6% risk** | 4 (26.7) | 2 (22.2) | 4 (50.0) |  |
| **17% risk** | 10 (66.7) | 5 (55.6) | 4 (50.0) |  |
| **45% risk** | 0 | 0 | 0 |  |

**p*-values were calculated using χ^2^ tests. Abbreviation: EORTC, European Organisation for Research and Treatment of Cancer.
