# Supplementary figures and images for: A Molecular Signature Determines the Prognostic and Therapeutic Subtype of Non-Muscle-Invasive Bladder Cancer Responsive to Intravesical Bacillus Calmette-Guérin Therapy
Source: Int J Mol Sci. 2021 Feb 1;22(3):1450. doi: 10.3390/ijms22031450 (PMC7867154; doi:10.3390/ijms22031450)

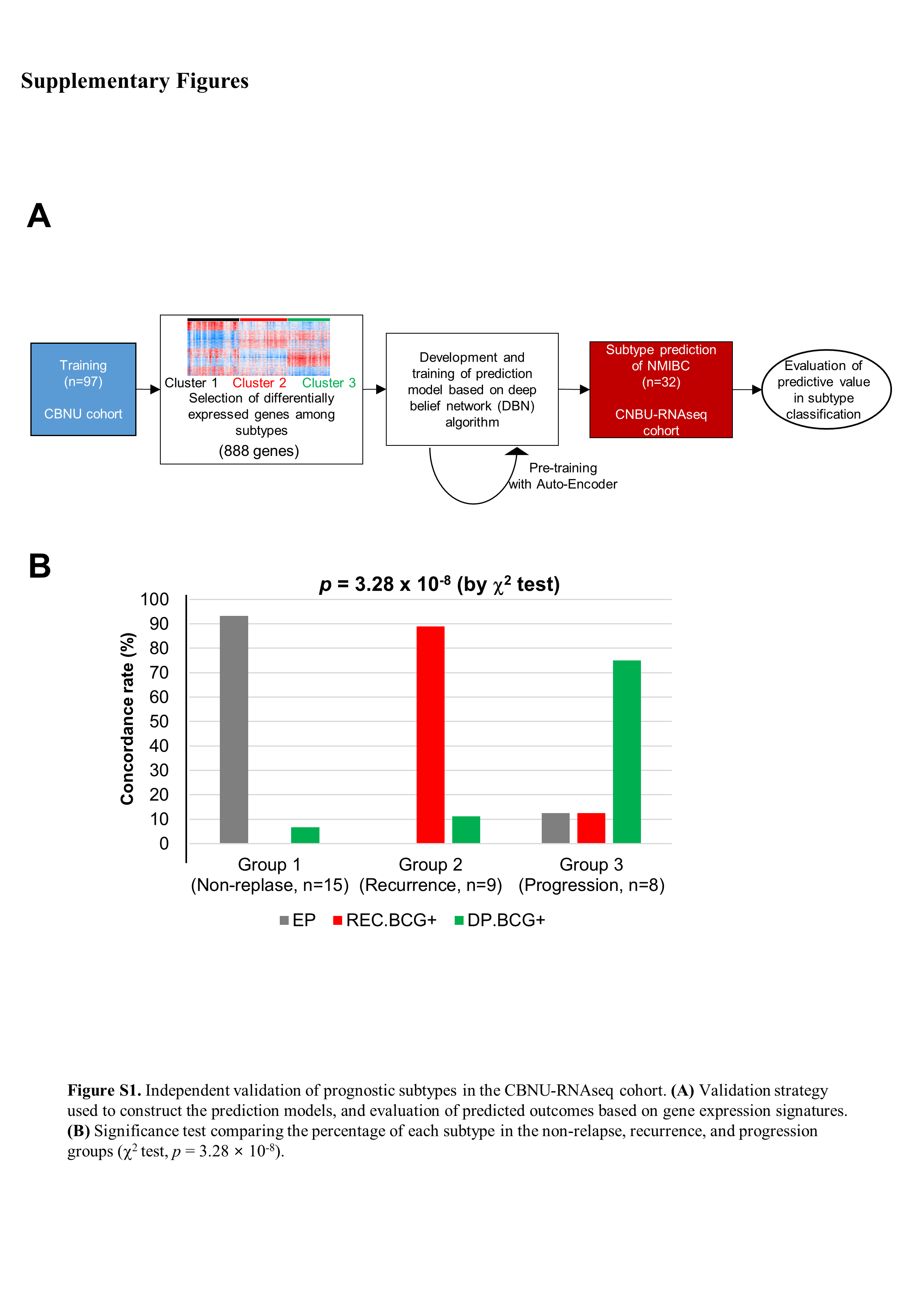

Supplement: Supplementary file 1 [file ijms-22-01450-s001.zip › Figure_S1.tif]

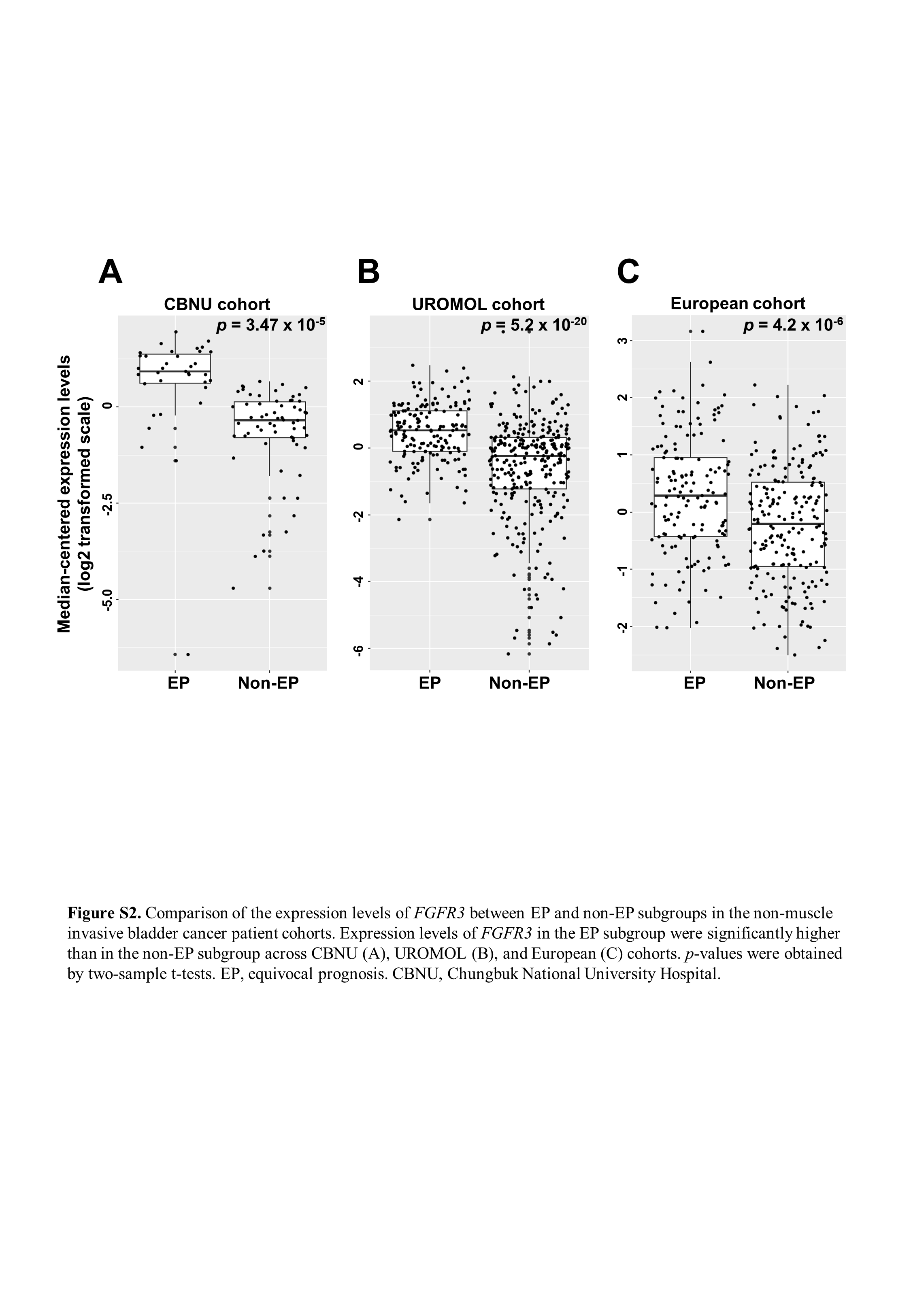

Supplement: Supplementary file 1 [file ijms-22-01450-s001.zip › Figure_S2.tif]

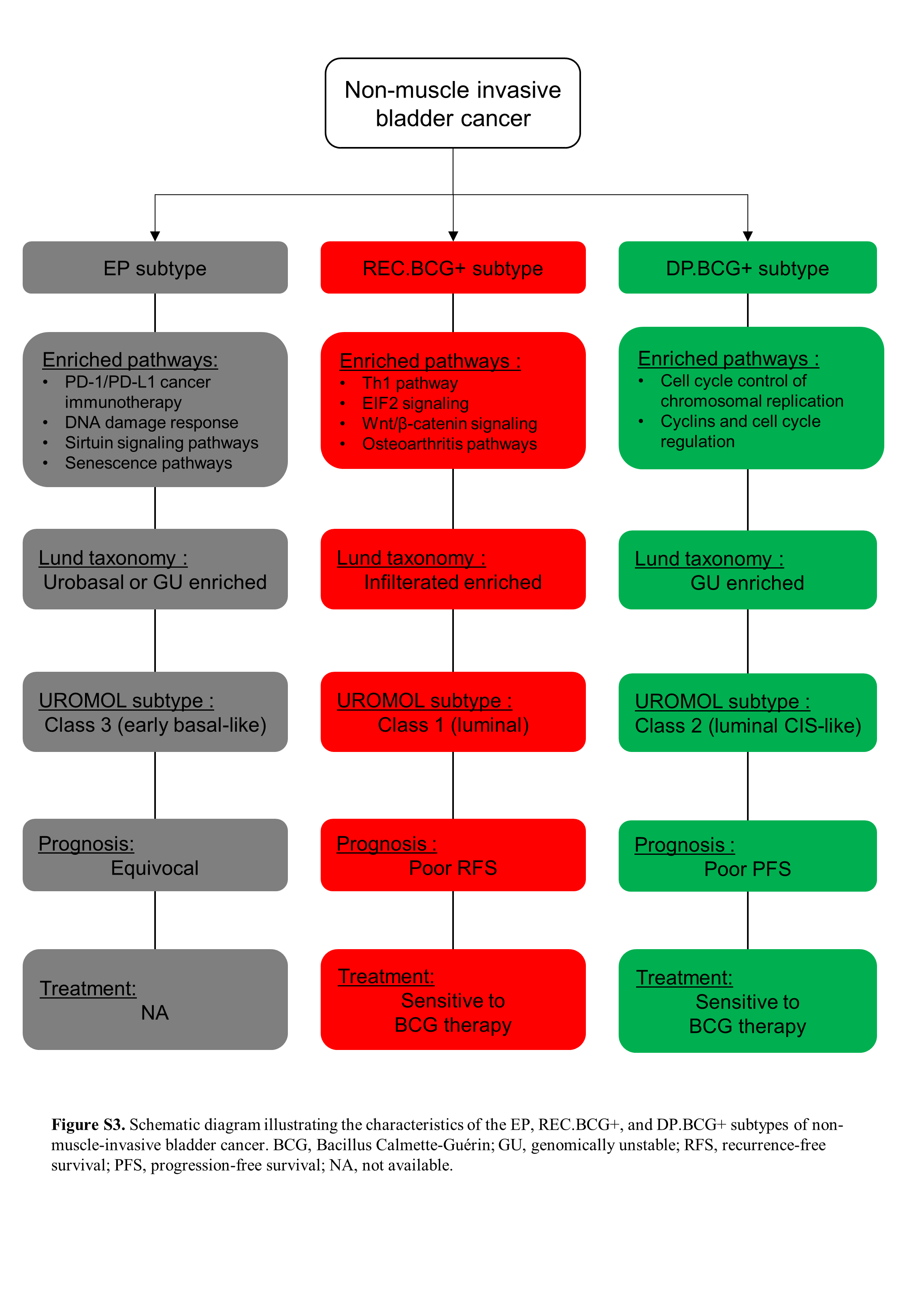

Supplement: Supplementary file 1 [file ijms-22-01450-s001.zip › Figure_S3.tif]

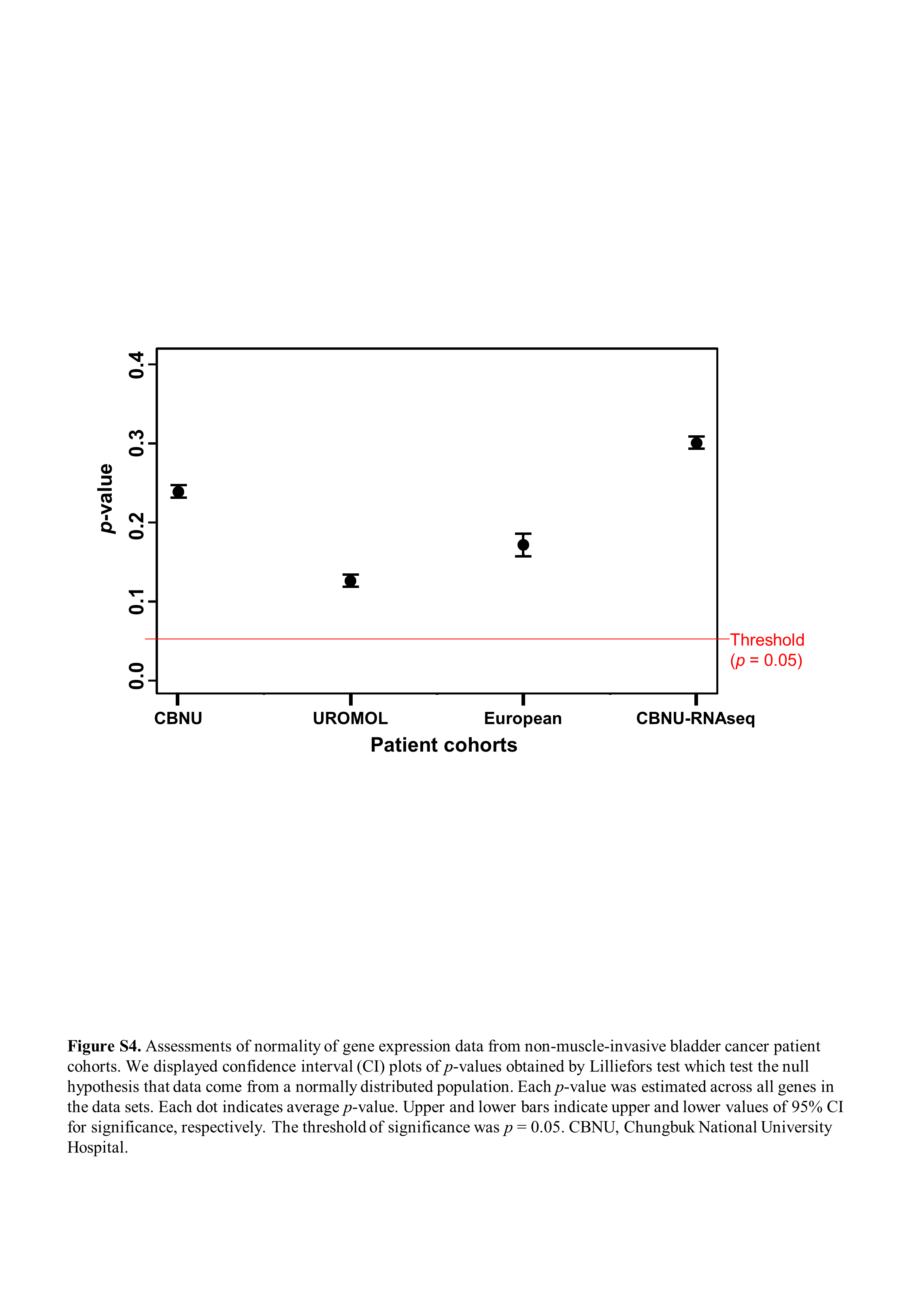

Supplement: Supplementary file 1 [file ijms-22-01450-s001.zip › Figure_S4.tif]
